# Comparative analysis of postoperative inflammation and pain: robot-assisted versus single-incision laparoscopic surgery for right-sided colon cancer

**DOI:** 10.1007/s00464-025-11970-4

**Published:** 2025-07-09

**Authors:** Yasuhiro Ishiyama, Yasumitsu Hirano, Sohei Akuta, Yume Minagawa, Akihito Nakanishi, Yusuke Nishi, Hisashi Hayashi, Takatsugu Fujii, Hirofumi Sugita, Chikashi Hiranuma

**Affiliations:** Department of Gastroenterological Surgery, Saitama Medical University International Medical Center, 1397-1 Yamane, Hidaka, Saitama Japan

**Keywords:** Colorectal cancer, Robot-assisted surgery (RAS), Single-incision laparoscopic surgery (SILS), Propensity score matching (PSM)

## Abstract

**Background:**

In recent years, surgical approaches for treating colorectal cancer have shifted from open surgery to laparoscopic surgery and, more recently, to robot-assisted surgery (RAS), yet their relative impact on postoperative inflammation and pain remains unclear. Therefore, this study aimed to compare the postoperative C-reactive protein/albumin ratio (CRA) and Numerical Rating Scale (NRS) scores between RAS and single-incision laparoscopic surgery (SILS) for right-sided colon cancer to assess which surgical modality might be more minimally invasive.

**Methods:**

We retrospectively analyzed 180 patients who underwent surgical resection for right-sided colon cancer at Saitama Medical University International Medical Center (Hidaka, Saitama, Japan) between March 2021 and October 2024. Among these patients, 80 underwent RAS (RAS group) and 100 underwent SILS (SILS group). Propensity score matching (PSM) was applied, and 67 patients from each group were selected for comparison. Primary outcomes were the CRA and pain intensity on the NRS on postoperative days (POD) 1 and 3. Secondary outcomes included operative time, blood loss, and major complications (Clavien–Dindo > II).

**Results:**

After PSM, there were no significant differences in the patient background variables, including the preoperative CRA, between the groups. The operative time was significantly shorter in the SILS group than in the RAS group (SILS vs. RAS: 157 vs. 193 min, *p* < 0.001). Although the CRA did not differ on POD 1 and 3, the SILS group had a significantly higher CRA than the RAS group. The RAS group had a significantly lower NRS score on POD 1.

**Conclusion:**

For right-sided colon cancer surgery, SILS involves fewer and smaller incisions and a shorter operative time. However, RAS was associated with a lower early postoperative inflammatory response and pain in this PSM comparison. The refined precision and reduced tissue manipulation offered by robotic systems may explain these differences.

Colorectal cancer is a leading cause of cancer-related mortality in developed countries, including Japan, with increasing incidence rates [1]. Right-sided colon cancer accounts for approximately 40% of all colorectal cancers, and treatment strategies range from endoscopic interventions to surgical resection [2]. In recent years, surgical approaches for treating colorectal cancer have shifted from open surgery to laparoscopic surgery and, more recently, to robot-assisted surgery (RAS). RAS offers several potential advantages, including a three-dimensional surgical view, tremor filtration, and articulating instruments that permit precise surgery. Although these factors may reduce the physical burden on surgeons and facilitate meticulous lymph node dissection, concerns have been raised regarding the higher costs and the potentially longer operative times associated with the procedure [3, 4]. Moreover, multiple reports have indicated no significant differences in long-term outcomes between RAS and conventional laparoscopic surgery [4].

Single-incision laparoscopic surgery (SILS) is a minimally invasive approach aimed at reducing the number and size of incisions, often through a single port placed in the umbilicus. This technique has potential benefits in terms of cosmesis and pain reduction [5, 6], with several studies demonstrating long-term outcomes comparable with those of conventional laparoscopic surgery [7].

Some reports have suggested that RAS causes relatively less tissue trauma and lower inflammatory responses owing to its more refined manipulation [8, 9]. Conversely, SILS requires only a single incision, which may result in higher patient satisfaction by minimizing surgical wounds [10]. While comparisons between conventional laparoscopic surgery and RAS have been widely reported, only a few studies have directly compared RAS and SILS with respect to the postoperative inflammatory markers and pain.

Therefore, this study aimed to compare the postoperative C-reactive protein/albumin ratio (CRA) and Numerical Rating Scale (NRS) scores between RAS and SILS for right-sided colon cancer to assess which surgical modality might be more minimally invasive.

## Patients and methods

### Patients

**Fig. 1:**
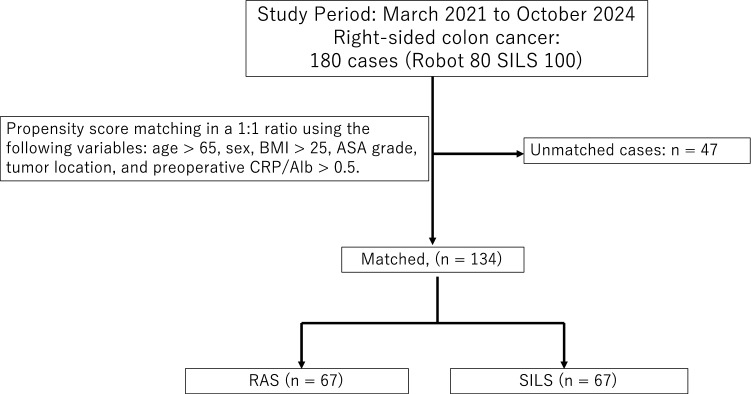
Flowchart

We retrospectively analyzed 180 patients who underwent surgical resection for right-sided colon cancer at Saitama Medical University International Medical Center (Hidaka, Saitama, Japan) between March 2021 and October 2024. Among these patients, 80 underwent RAS (RAS group) and 100 underwent SILS (SILS group). Propensity score matching (PSM) was applied, and 67 patients from each group were selected for comparison (Fig. 1). Inclusion criteria: Patients were eligible if they (1) were aged ≥ 18 years; (2) had histologically confirmed adenocarcinoma of the right colon; and (3) underwent curative standard ileocecal resection or right hemicolectomy with systematic lymphadenectomy, performed either through SILS or RAS using the da Vinci Xi platform. Exclusion criteria: Patients were excluded if they (1) had synchronous or metachronous malignancies in other organs, (2) required emergency surgery for bowel obstruction or perforation, or (3) had undergone stoma construction (diverting or permanent).

### Surgeon

Although the choice of surgical approach was primarily based on surgeon preference, a total of eight surgeons—all certified by the Endoscopic Surgical Skill Qualification System of the Japan Society for Endoscopic Surgery—performed both RAS and SILS procedures. Each surgeon had experience with $>$ 50 laparoscopic colorectal resections, minimizing potential bias due to differences in technical skills. When a dedicated robotic console was available, they tended to select RAS; conversely, SILS was chosen if the console was occupied or another higher-priority robotic case had already been scheduled [11].

### Surgical procedure

#### RAS

Patients were placed in the supine position with the left side tilted downward and the head slightly lowered. A 3-cm transumbilical incision was made, through which an EZ access device was inserted. Four additional 8-mm ports were placed in the lower abdomen and surgery was performed using the da Vinci Xi system, typically with 5 ports. An intracorporeal medial-to-lateral approach was used. After identifying the duodenum, the superior mesenteric vein (SMV) was exposed, and the ileocolic artery (ICA) and vein (ICV) were ligated. Dissection was continued cranially along the SMV, followed by division of the middle colic artery (MCA) and accessory right colic vein. After lymph node dissection was completed, the colon was fully mobilized from the retroperitoneum to the hepatic flexure. The greater omentum was divided near the duodenum toward the hepatic flexure. The specimen was extracted through the umbilical incision and an extracorporeal anastomosis was performed (functional end-to-end anastomosis).

#### SILS

Patients were also placed in the supine position with the left side tilted downward and the head slightly lowered. A 3-cm transumbilical incision was made and an EZ Access device was used to insert with 3 ports (1 12-mm and 2 5-mm ports). The surgical steps (medial-to-lateral approach, lymph node dissection, and extracorporeal anastomosis) were performed in a manner similar to the RAS group, except that all instruments were manipulated through a single port.

### Data collection and outcomes

We retrospectively collected data on demographic and clinical variables, including age, sex, body mass index (BMI), the American Society of Anesthesiologists (ASA) classification, tumor location, clinical stage, operative time, estimated blood loss, postoperative complications (Clavien–Dindo grade > 2) [12], and postoperative hospital stay from electronic medical records.

### Inflammatory and pain assessments

Routine blood tests were conducted preoperatively and on postoperative days (PODs) 1 and 3. We measured and compared the postoperative CRA (CRP/Alb ratio) on PODs 1 and 3 between the groups. Postoperative pain was assessed using the NRS on PODs 1 and 3.

#### NRS

Nurses assessed pain at the bedside using the NRS (0–10) before and after surgery.

### Sample size calculation

A clinically meaningful difference in the NRS was defined as ≥ 1.0 points [14]. Assuming a standard deviation (*σ*) of 2.0 for the NRS, an alpha of 0.05 (two-sided), and a power (1–*β*) of 0.80, a sample size model for two-group comparisons (*t* test) suggested that 65 patients per group (130 in total) would be sufficient.

### Statistical analysis

All statistical analyses were performed using JMP Pro 17 software (SAS Institute, Cary, NC, USA). Continuous variables were compared with the unpaired Student’s *t* test or the Mann–Whitney *U* test, as appropriate, and categorical variables with the *χ*^2^ test. Two-sided *P* values < 0.05 were considered statistically significant.

To minimize baseline imbalances, PSM was performed using age > 65 years, sex, BMI > 25 kg/m^2^, ASA physical status, and preoperative CRA > 0.05 as covariates. A BMI cutoff of 25 kg/m^2^ reflects the Japanese definition of obesity, which follows the WHO Western Pacific Region and the Japan Society for the Study of Obesity guidelines. Thus, it differs from the global WHO criterion of 30 kg/m^2^ [13]. The age threshold of 65 years corresponds to the WHO’s definition of older adults [14]. The CRA cutoff of 0.05 represented the median value for the entire cohort.

Patients in the RAS group were matched 1:1 with those in the SILS group using the nearest-neighbor Mahalanobis distance method, with a caliper width of 0.05 of the propensity score, yielding 67 well‑balanced pairs for subsequent analyses.

## Results

### Patient characteristics before and after PSM

Before PSM, no significant differences were observed in age, sex, BMI, ASA classification, or tumor location between the two groups. However, preoperative CRP/Alb levels were significantly higher in the SILS group. After PSM, there were no significant differences in the patient background variables, including the preoperative CRA, between the groups. The standardized mean differences (SMD) of main covariates after matching were as follows: Age > 65 (SMD =  − 0.42), sex (SMD =  − 0.03), BMI > 25 (SMD = 0.20), ASA classification (SMD =  − 0.26), and CRP/Alb > 0.05 (SMD =  − 0.12). Except for age and ASA classification, the SMD was < 0.2, indicating a good balance (Table 1).

**Table 1:**
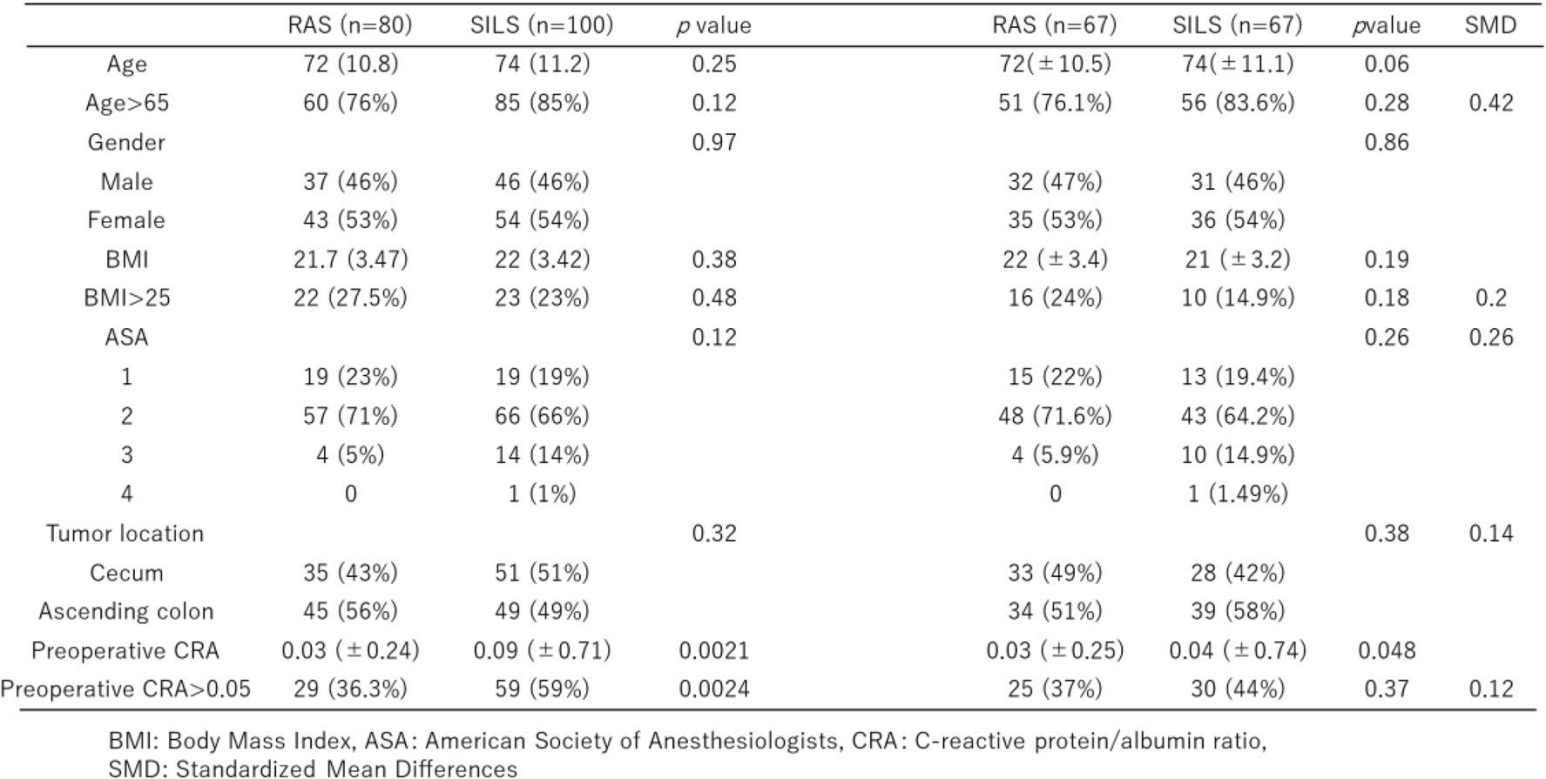
Patient characteristics before and after propensity score matching

### Operative outcomes after PSM

The operative time was significantly shorter in the SILS group than in the RAS group (SILS vs. RAS: 157 vs. 193 min, *p* < 0.001). No significant differences were observed in postoperative complications (Clavien–Dindo score > 2), blood loss, length of hospital stay, or need for blood transfusion. Pathologic findings did not differ significantly between the 2 groups (Table 2).

**Table 2:**
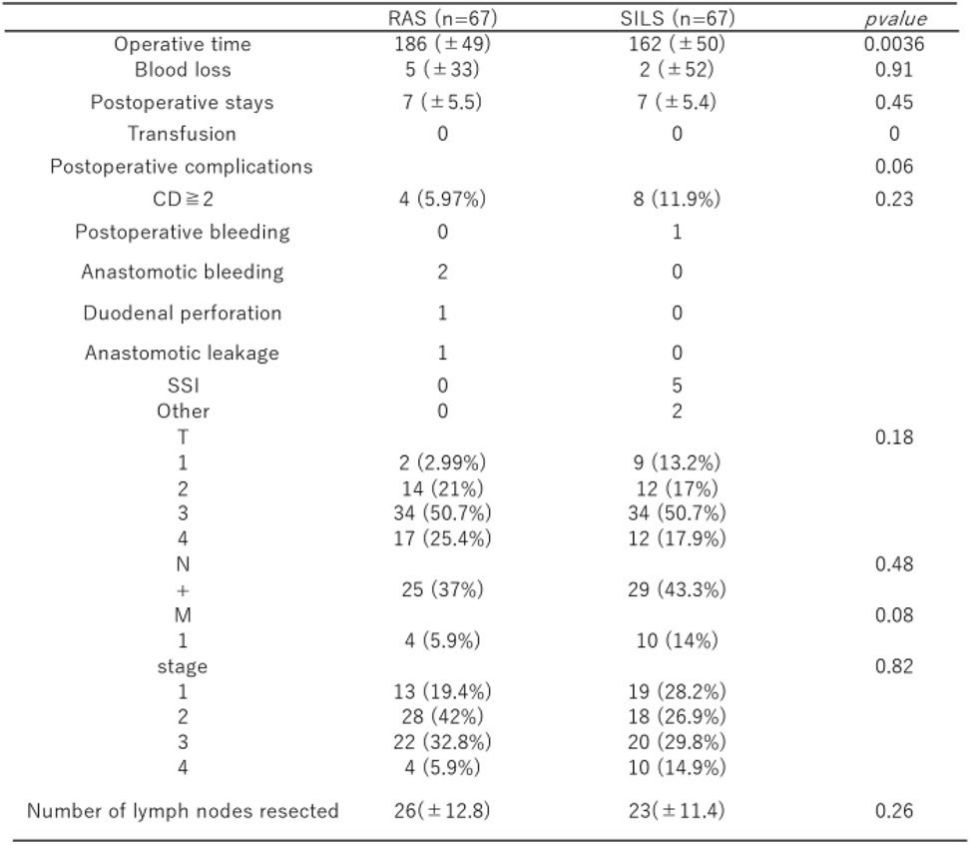
Operative outcomes before and after propensity score matching

*CD* Clavien–Dindo, *SSI* surgical site incision

#### CRA

After PSM, there were no significant between-group differences in the CRA on POD 1. However, on POD 3, the SILS group had a significantly higher CRA than the RAS group (Table 3).

**Table 3:**
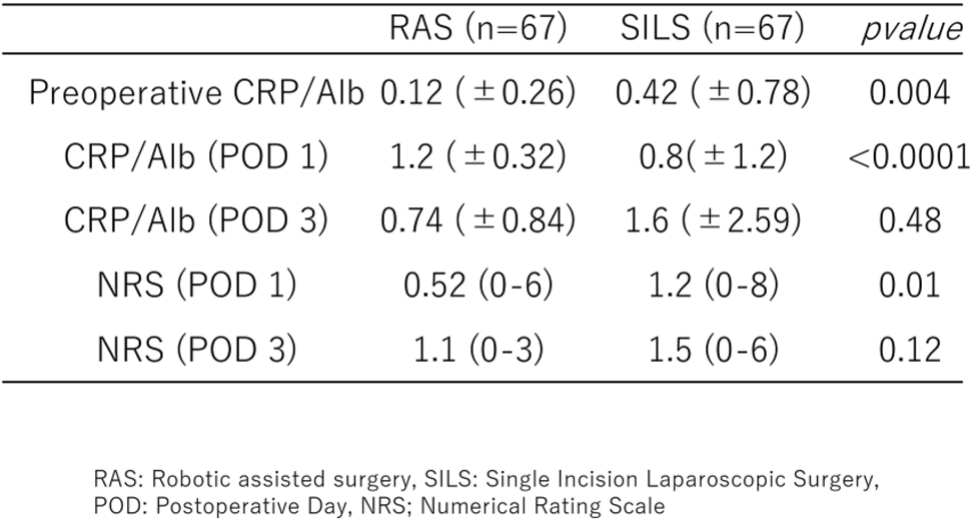
CRP/albumin ratio and numerical rating scale scores before and after surgery: Robotic-assisted surgery vs. Single-incision laparoscopic surgery

#### NRS

After PSM, the RAS group had a significantly lower NRS score on POD 1. However, on POD 3, no statistically significant difference was observed between the 2 groups (Table 3).

### Association between the CRA and postoperative outcomes

Patients who developed postoperative complications (Clavien–Dindo grade > II) had significantly higher CRA values on POD 3 than those without complications. Postoperative hospital stays longer than 7 days showed significantly higher CRA values on POD1 and POD 3 than those discharged within 7 days (Table 4).

**Table 4:**
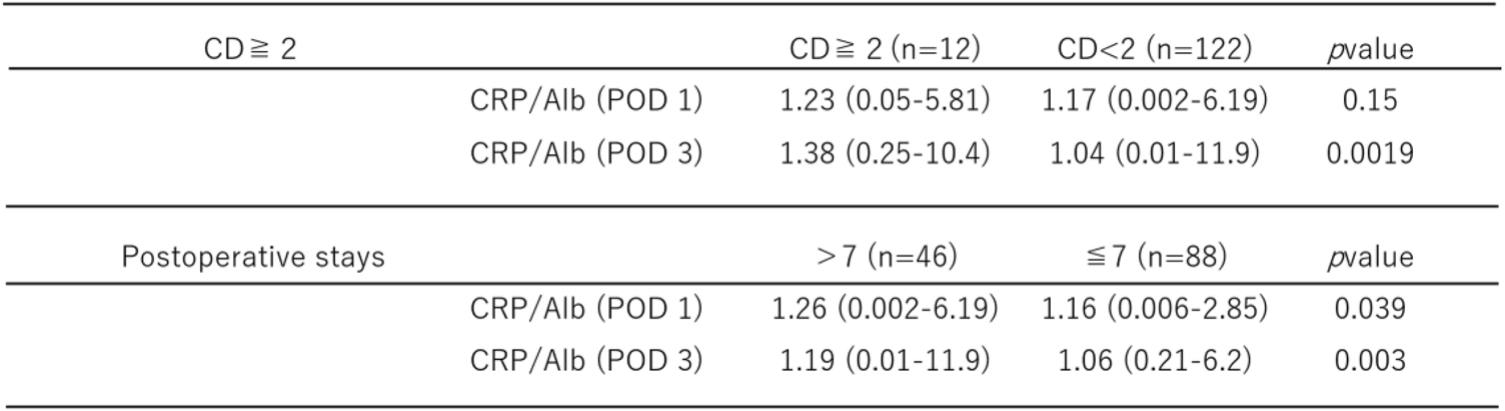
Association between CRP/albumin ratio and postoperative outcomes

## Discussion

In this retrospective study, we compared the short-term outcomes and invasiveness between RAS and SILS for the treatment of right-sided colon cancer. After adjusting for confounders using PSM, the operative time was significantly shorter in the SILS group (approximately 34 min), which could be clinically meaningful for operating room turnover and total anesthesia time. However, the SILS group showed a higher CRA on POD 3 (0.07), exceeding the commonly reported cutoff of 0.05, which is often associated with an increased risk of infection or inflammatory complications [15]. Moreover, on POD 1, the RAS group’s NRS score was approximately 1.2 points lower, surpassing the minimal clinically important difference of 1.0 on the 0–10 pain scale [16]. Therefore, despite the shorter operative time and fewer incisions, SILS may be associated with a slightly higher inflammatory response and earlier onset of postoperative pain than RAS. This finding suggests that RAS may be a less invasive treatment option for some patients.

The CRA reflects both elevated CRP and decreased albumin levels, providing a sensitive marker of acute inflammatory response and tissue trauma [15, 17]. The CRA is an integrated marker that reflects both the acute-phase response, represented by elevated CRP levels, and the nutritional status, indicated by decreased albumin levels. In the field of gastrointestinal surgery, its utility has been reported as a predictive indicator of postoperative complications and recovery trajectories [18] and [19, 20]. In the present study as well, an elevated CRP level was associated with both Clavien–Dindo grade > II complications and prolonged postoperative hospital stay. Robot-assisted procedures may reduce minute vascular or tissue injuries owing to more precise manipulation [19, 20], potentially reducing postoperative inflammation. In this study, the higher CRA observed in the SILS group may have resulted from the concentration of instruments through a single port, possibly leading to excessive tension or mechanical stress at the incision site.

Additionally, although SILS offers cosmetic benefits by limiting the number of incisions, several reports have indicated that intense traction through a single port can paradoxically increase pain, at least in the early postoperative period [21]. Our data support this notion, as the SILS group had higher pain scores on POD 1, although no difference was observed on POD 3. Recent studies have also reported the utility of the da Vinci SP system for colorectal cancer, suggesting that it may reduce postoperative pain more effectively than the Xi system, potentially due to the decreased abdominal wall impact from the robotic remote center [22, 23]. Furthermore, postoperative pain has been shown to significantly influence patient satisfaction after minimally invasive procedures, and reduced pain may bias patients to choose robot-assisted surgery in future [24, 25].

Although large-scale randomized controlled trials identified no significant long-term oncologic differences between RAS and standard laparoscopic surgery [3, 8, 9], other analyses have indicated better early postoperative pain control and faster recovery with RAS. Meanwhile, meta-analyses of SILS and conventional laparoscopic surgery reported comparable short-term complications and oncologic outcomes, albeit with increased technical difficulty and a prolonged operative time [6, 26]. Nonetheless, higher patient satisfaction at the 1-year follow-up with SILS underscores the importance of cosmesis as a key factor in patient-centered surgical outcomes [10]. Although numerous studies have compared standard laparoscopic surgery with RAS, the present study evaluated the potentially less invasive SILS versus RAS to more clearly assess the additional benefits of RAS. Constrastingly, adopting the da Vinci SP system could combine the cosmetic advantages of a single incision with the precise, minimally invasive manipulation of the robotic system [22, 23].

This study has several limitations. First, this was a single-center, retrospective study with inherent selection bias, although we used PSM to minimize confounding factors. Second, pain assessment relied on patient-reported NRS scores, which may have been subjective. We did not measure levels of IL-6 or other potentially objective inflammatory markers. Third, variables such as cost, patient-reported outcomes, and surgeon learning curves were not formally assessed. These factors may influence operative time, inflammatory response, and overall outcomes [27]. Nevertheless, all participating surgeons had prior experience with over 50 cases of laparoscopic colorectal resection, thereby minimizing the potential impact of surgeon-related bias. Fourth, multiple comparisons were performed for inflammatory and pain-related outcomes across different postoperative days, which may increase the risk of type I error. However, we applied Bonferroni correction to control for this, and the significant differences in CRP/Alb ratio on POD3 and NRS on POD1 remained statistically significant, supporting the robustness of our findings.

## Conclusion

For right-sided colon cancer surgery, SILS involves fewer and smaller incisions and a shorter operative time. However, RAS was associated with a lower early postoperative inflammatory response and pain in this PSM comparison. The refined precision and reduced tissue manipulation offered by robotic systems may explain these differences. Ultimately, the selection of the optimal surgical approach must consider patient anatomy, tumor characteristics, surgeon expertise, and each institution’s resources. Large-scale prospective trials, including randomized controlled studies, are warranted to define the role of RAS and SILS in right-sided colon cancer.
